# Evaluation of job satisfaction of practice staff and general practitioners: an exploratory study

**DOI:** 10.1186/1471-2296-12-137

**Published:** 2011-12-12

**Authors:** Katja Goetz, Stephen M Campbell, Jost Steinhaeuser, Bjoern Broge, Sara Willms, Joachim Szecsenyi

**Affiliations:** 1 Department of General Practice and Health Services Research, University of Heidelberg, Germany; 2 National Primary Care Research and Development Centre, University of Manchester, UK; 3 AQUA-Institute for Applied Quality Improvement and Research in Health Care, Goettingen, Germany

## Abstract

**Background:**

Primary care teams' job satisfaction is an important issue in quality of care. The purpose of our study was to evaluate the job satisfaction of general practitioners (GPs) and non-physician staff and to explore the elements that may impact on overall job satisfaction for GPs and non-physician staff separately.

**Methods:**

The study was based on data from the European Practice Assessment and used an observational design. Job satisfaction was measured with the 10-items Warr-Cook-Wall questionnaire with 7-point-Likert scales. Job satisfaction of GPs and non-physician staff was compared and impact on overall job satisfaction was analysed with stepwise linear regression analyses for both samples separately.

**Results:**

The study population consisted of 2878 non-physician staff (mean age: 38 years) and 676 GPs (mean age: 50 years). The actual mean working time per week of GPs was 50.0 hours and of practice staff 26.0 hours. Both were satisfied with colleagues and fellow workers (mean = 5.99 and mean = 6.18 respectively) and mostly dissatisfied with their income (mean = 4.40 and mean = 4.79 respectively). For GPs the opportunity to use their abilities (β = 0.638) and for non-physician staff recognition for their work (β = 0.691) showed the highest scores of explained variance (R^2 ^= 0.406 and R^2 ^= 0.477 respectively) regarding overall job satisfaction.

**Conclusions:**

Non-physician staff evaluate their job satisfaction higher than GPs except recognition for work. Job satisfaction of members of primary care teams is important because poor satisfaction is associated with suboptimal healthcare delivery, poor clinical outcomes and higher turnover of staff.

## Background

Primary care teams' job satisfaction is an important issue in quality of care for a number of reasons. Poor satisfaction is associated with suboptimal healthcare delivery and poor clinical outcomes, for instance due to adverse events and reduced patient adherence [1,2]. Job dissatisfaction is a major cause of nurses' turnover and a shortage of non-physician personnel [3,4]. Another negative consequence of high turnover is a loss of continuity of care [5]. For the healthcare workers themselves, lowered job satisfaction is also associated with higher levels of stress and burnout [6,7]. Furthermore, it may affect patient satisfaction with care [8]. It is known that working conditions have an important impact on job satisfaction and a high workload is associated with a lower performance at primary care practices [9]. Evidence suggests that many health care workers are very dissatisfied with their income and overall working conditions [4].

Feelings of job dissatisfaction and job stress are problems shared by nurses as well as general practitioners (GPs) in western countries [10,11]. The Commonwealth Fund surveyed GPs from 7 countries to compare different aspects of work and showed that German GPs have the highest workload and were most dissatisfied compared to their colleagues from other countries [11]. Germany is based on a Social Security Health system and is funded by means of earmarked premiums. Over 60.000 GPs were registered in 2009 in Germany according to the National Association of Statutory Health Insurance Physicians. In Germany 94% of all GPs are self-employed. The majority (64%) works in consist of solo practices [12]. Data about practice location are not consistent. In studies performed in Germany participants rate themselves as either rural or urban located. More objective measurements are not implemented yet [13]. Furthermore, GPs in Germany do not function as a strict gatekeeper as patients have free access to ambulatory specialist services too.

Job satisfaction of GPs decreases with a higher number of hours worked and low income. It increases when there is more contact with other colleagues and greater job variety [14]. One study in Australian compared job satisfaction of physician and non-physician staff and showed that there is overall satisfaction with colleagues but dissatisfaction with income in both groups of employees [15].

The concept of job satisfaction was defined by Herzberg and Mausner [16]. They distinguished between intrinsic satisfaction such as recognition, the work tasks themselves and the level of or responsibility on the one hand, and extrinsic factors like working conditions, company policy or salary, which influence job satisfaction on the other hand [16].

The aim of this study was to evaluate the job satisfaction of German GPs and their non-physician staff separately. The research questions of the study were: 1) How do GPs and non-physician staff evaluate their job satisfaction? 2) Which elements are associated with overall job satisfaction for the group of GPs and the one of non-physician staff?

## Methods

The assessment of job satisfaction among healthcare workers in general practice is one component of the European Practice Assessment (EPA) [17]. EPA consists of a set of validated quality indicators for external and internal assessment, a patient survey of satisfaction with care, a staff job satisfaction survey, an outreach visit by a trained visitor, structured feedback and a team-meeting in the practice [17].

### Design and participants

This observational study was based on a job satisfaction survey. This study used data from the routine implementation of quality management in ambulatory care in Germany, in line with regulations outlined by law in the social code book V and the directives on quality management of the Federal Joint Committee [18]. There are different quality management systems available for ambulatory care are in Germany such as QEP, ISO 9001:2008, KTQ, EFQM, KPQ and EPA [19]. Practices can choose on their own among these different systems. Data were collected from 676 practices, which had used the European Practice Assessment methodology [17]. The subjects involved in the study included 2947 non-physician staff members (consisting of 2332 physician assistants, 101 nurses, 69 practice managers, 39 secretarial assistants, 337 other workers and 69 with unknown profession) and 676 practice principal physicians of general practices in Germany who have undertaken the EPA.

### Measures

All participants completed the same written questionnaire, which was returned in a pre-paid envelope to the AQUA-institute domiciled in Goettingen, Germany. Data collection took place between May 2004 and September 2007. The questionnaire included the following items:

1) Individuals were asked to complete questions about gender and age, as well as how many hours a week they worked at their practice, and, 2) Job satisfaction was measured with the German validated version of the Warr-Cook-Wall (WCW) job satisfaction scale developed by Warr et al. [20]. The WCW instrument measures overall job satisfaction and satisfaction with nine aspects of work (amount of variety in job, opportunity to use abilities, freedom of working method, amount of responsibility, physical working condition, hours of work, income, recognition for work, and colleagues and fellow workers), with each item rated on a 7-point Likert scale (1 = extreme dissatisfaction to 7 = extreme satisfaction). A higher overall mean score indicates higher job satisfaction.

### Data analysis

The analyses were performed using SPSS version 18.0 (SPSS Inc., Chicago IL, USA). Differences between physician and non-physician staff were analysed using Student's t test for continuous variables as appropriate and Chi-square test for categorical variables. The intraclass correlation coefficient (ICC) was calculated to determine consistency between physician and non-physician staff regarding the evaluation of job satisfaction. Furthermore, a descriptive analysis was performed concerning the overall job satisfaction and nine other items of the job satisfaction scale separated for physicians and non-physician staff. Statistical significance of group comparisons for the whole study population and for full-time staff was calculated with Students' unpaired t-test with list wise exclusion of missing data. Means and standard deviations of these items are reported in the results section. Afterwards stepwise linear regression analyses were performed for physicians as well as for non-physician staff separately. Overall job satisfaction was the outcome variable while other elements of satisfaction with work and some characteristics of participants (age, sex, mean weekly working time, location and mode of practice) were potential predictors. An alpha level of p < 0.05 was used for tests of statistical significance. However, as this was an exploratory analysis, *p *values can only be descriptive in nature.

### Ethics

Ethical approval was not necessary. Our study used data from the routine implementation of quality management in ambulatory care in Germany, according to the social code book V and the directives on quality management of the Federal Joint Committee. The questionnaires were completed anonymously. No additional information or data from patients or staff were requested to perform this study.

## Results

### Description of the study sample

The response rate of the job satisfaction survey was 100%, because it is based on a quality management programme and therefore mandatory for the participants. There were 676 practice principals who completed the EPA between May 2004 and September 2007. Out of 3273 staff member questionnaires handed out, 2947 were returned, giving a response rate of 90%. However, 69 individuals with unknown profession were excluded from the sample of non-physician staff. Therefore, in all we involved 2878 non-physician staff members out of 676 primary care practices in this study.

Table 1 presents the individual characteristics of participants. Physicians and non-physician staff showed significant differences in all three individual characteristics. More than 70% of the physicians were male. The mean age was 50.0 years (SD = 7.6) and mean weekly work time was 50.6 hours (SD = 12.8). The majority of the non-physician sample was female (97.2%). The mean age of that group was 38.3 years (SD = 12.2). The mean weekly work time was 26.0 hours (SD = 12.5). The sample consisted of 46.9% solo practices and over 51.8% practices of our sample were located in rural areas in Germany.

**Table 1:** Characteristics of the participants

	Physician (n = 676)	Non-physician staff (n = 2878)	p-Value^#^
Age [mean (SD)]	50.13 (7.59)	38.31 (12.24)	< 0.001
Sex (male)	70.80%	2.80%	< 0.001
Mean weekly working time [mean (SD)]	50.63 h (12.85)	26.03 h (12.25)	< 0.001

SD standard deviation

^#^Statistical significance p < 0.05

The design effect calculated by ICC for physician and non-physician staff regarding the elements of job satisfaction was negligible. For example physical working condition showed an ICC of 0.007 (p = 0.431), freedom of working method an ICC of 0.013 (p = 0.367) and amount of responsibility an ICC of -0.005 (p = 0.556).

### Evaluation of job satisfaction separated for physicians and non-physician staff

Questions about job satisfaction were completed by 643 GPs (95.1%) out of the 676 respondents and from 2634 non-physician staff members (91.5%) out of the 2878 respondents. GPs and non-physician staff were satisfied with 'colleagues and fellow workers' (mean = 5.99 and mean = 6.18 respectively) and mostly dissatisfied with their 'income' (mean = 4.40 and mean = 4.79 respectively). Non-physician staff had higher levels of satisfaction in questions about job satisfaction with the exception of 'recognition for work'. Details are given in Table 2.

**Table 2:** Job satisfaction of all practice staff for each of the 10 items on the Warr-Cook-Wall (WCW) job satisfaction scale*

WCW items	Physician (n = 643) Mean (SD)	Non-physician staff (n = 2634) Mean (SD)	p-Value^#^
1. Amount of variety in job	5.70 (1.23)	5.94 (1.15)	< 0.001
2. Opportunity to use abilities	5.37 (1.53)	5.82 (1.17)	< 0.001
3. Freedom of working method	5.66 (1.41)	5.82 (1.23)	0.005
4. Amount of responsibility	5.64 (1.36)	5.92 (1.34)	< 0.001
5. Physical working condition	5.19 (1.45)	5.63 (1.25)	< 0.001
6. Hours of work	4.43 (1.67)	5.75 (1.32)	< 0.001
7. Income	4.40 (1.60)	4.79 (1.65)	< 0.001
8. Recognition for work	5.57 (1.62)	5.41 (1.49)	0.017
9. Colleagues and fellow workers	5.99 (1.00)	6.18 (1.02)	< 0.001
10. Overall job satisfaction	5.56 (1.12)	5.95 (1.05)	< 0.001

*Possible score for each item between 1 (extremely dissatisfied) and 7 (extremely satisfied).

^#^Statistical significance p < 0.05

SD standard deviation

Table 3 shows the evaluation of job satisfaction for the full-time staff. Out of 676 GPs in our study population 523 GPs (77.4%) worked 38 hours and more per week. The corresponding number for non-physician staff 743 (25.8%). Compared to the whole sample described in Table 2, full-time GPs and non-physician staff were equally satisfied with 'colleagues and fellow workers' (mean = 5.99 and mean = 6.13 respectively) and mostly dissatisfied with their 'income' (mean = 4.37 and mean = 4.49 respectively). Moreover, GPs were also dissatisfied with their 'hours of work' (mean = 4.21). The results are also depicted in Table 3.

**Table 3:** Job satisfaction of full-time practice staff for each of the 10 items on the Warr-Cook-Wall (WCW) job satisfaction scale*

WCW items	Physician (n = 523) Mean (SD)	Non-physician staff (n = 743) Mean (SD)	p-Value^#^
1. Amount of variety in job	5.66 (1.24)	5.78 (1.24)	0.086
2. Opportunity to use abilities	5.31 (1.55)	5.76 (1.19)	< 0.001
3. Freedom of working method	5.60 (1.43)	5.67 (1.32)	0.331
4. Amount of responsibility	5.59 (1.38)	5.83 (1.24)	0.001
5. Physical working condition	5.09 (1.46)	5.40 (1.28)	< 0.001
6. Hours of work	4.21 (1.63)	5.44 (1.39)	< 0.001
7. Income	4.37 (1.58)	4.49 (1.70)	0.172
8. Recognition for work	5.52 (1.30)	5.35 (1.47)	0.035
9. Colleagues and fellow workers	5.99 (1.00)	6.13 (1.08)	0.021
10. Overall job satisfaction	5.52 (1.11)	5.80 (1.12)	< 0.001

*Possible score for each item between 1 (extremely dissatisfied) and 7 (extremely satisfied).

^#^Statistical significance p < 0.05

SD standard deviation

### Elements associated with overall job satisfaction separated for physicians and non-physician staff

The two regression models - for physicians and for non-physician staff - which are presented in Table 4 and Table 5 report only coefficients with statistically significances at the p < 0.05 level.

**Table 4:** Associations of individual characteristics and satisfaction of aspects of work of non-physician staff on overall job satisfaction (results of stepwise linear regression analysis, under specification of standardized beta coefficient, α = 5%)

	Step 1	Step 2	Step 3	Step 4	Step 5	Step 6	Step 7	Step 8	Step 9	Step 10	Step 11
Recognition for work	0.691	0.540	0.449	0.398	0.331	0.291	0.266	0.244	0.235	0.239	0.239
Amount of variety on job		0.356	0.297	0.241	0.229	0.171	0.165	0.162	0.158	0.158	0.164
Colleagues and fellow workers			0.252	0.236	0.211	0.196	0.190	0.192	0.187	0.187	0.185
Hours of work				0.202	0.164	0.147	0.139	0.122	0.119	0.116	0.117
Physical working condition					0.176	0.165	0.150	0.144	0.137	0.131	0.127
Opportunity to use abilities						0.149	0.117	0.111	0.101	0.105	0.104
Amount of responsibility							0.101	0.100	0.091	0.092	0.091
Income								0.071	0.072	0.067	0.068
Freedom of working method									0.051	0.049	0.054
Mean weekly working time										-0.034	-0.049
Age											-0.038

**Pseudo R^2^**	**0.477**	**0.581**	**0.628**	**0.659**	**0.677**	**0.688**	**0.693**	**0.696**	**0.697**	**0.698**	**0.699**

Only coefficients with statistically significances at the p < 0.05 level were reported.

**Table 5:** Associations of individual characteristics and satisfaction of aspects of work of GPs on overall job satisfaction (results of stepwise linear regression analysis, under specification of standardized beta coefficient, α = 5%)

	Step 1	Step 2	Step 3	Step 4	Step 5	Step 6	Step 7	Step 8	Step 9
Opportunity to use abilities	0.638	0.472	0.398	0.273	0.175	0.119	0.107	0.080	0.070
Hours of work		0.373	0.332	0.295	0.279	0.244	0.176	0.170	0.161
Colleagues and fellow workers			0.296	0.272	0.242	0.234	0.222	0.198	0.185
Freedom of working method				0.272	0.235	0.232	0.215	0.212	0.201
Amount of variety on job					0.220	0.229	0.230	0.215	0.204
Income						0.155	0.158	0.142	0.137
Physical working condition							0.137	0.136	0.129
Recognition for work								0.107	0.092
Amount of responsibility									0.085

**Pseudo R^2^**	**0.406**	**0.517**	**0.595**	**0.645**	**0.672**	**0.689**	**0.700**	**0.707**	**0.711**

Only coefficients with statistically significances at the p < 0.05 level were reported.

GP general practitioner

### Non-physician staff

Table 4 shows the stepwise regression analysis of individual characteristics and the elements of satisfaction with aspects of work on overall satisfaction for non-physician staff. A model with 11 steps was carried out and explained more than 70% (R^2^~ 0.70) of the variance of the dependent variable 'overall job satisfaction'. These were all nine elements of satisfaction with aspects of work, 'mean weekly working time' and 'age'. In the first step of the stepwise regression analysis the item 'recognition for work' showed the highest score (R^2 ^= 0.477) of explained variance. The variables sex, location and mode of practice were not included in the regression model since p > 0.05.

### Physicians

Table 5 shows the stepwise regression analysis of individual characteristics and elements of satisfaction with aspects of work on overall satisfaction for GPs. A model with nine steps was carried out and explained more than 71% (R^2^~ 0.71) of the variance of the dependent variable 'overall job satisfaction'. These were all nine variables of elements of satisfaction with aspects of work. In the first step of the stepwise regression analysis the item 'opportunity to use abilities' showed the highest score (R^2 ^= 0.406) of explained variance. The variables sex, gender, mean weekly working time, location and mode of practice were not included in the regression model since p > 0.05.

## Discussion

The purpose of our study was to evaluate the elements that have the main impact on overall job satisfaction separated for GPs and non-physician staff. Therefore, the job satisfaction of GPs and their non-physician staff at the same practices was observed. Our results showed that nearly all aspects of job satisfaction were rated higher by non-physician staff than by GPs. Only the item 'recognition for work' scored better for GPs than for non-physician staff. This difference remained for most questions of job satisfaction when only those who were full time were included in the analysis with expectation for amount of variety in job, freedom of working method and income. All participants, GPs and non-physician staff alike, were mostly dissatisfied with their income; GPs were more dissatisfied with their hours of work than non-physician staff; which might reflect the fact that the working hours of GPs in Germany are higher than in any other country in Europe [11,21].

It is not surprising that the item 'recognition for work' had the strongest association with more than 47% of explained variance of non-physician staff's overall job satisfaction. However, this is higher than in previous research [22]. This study also showed that being appreciated for high performance was most important to job satisfaction of non-physician staff [22]. This is important because recognition of performance facilitates team work. Work satisfaction of primary care teams correlates positively with higher scores for outcome quality measures [23].

The strongest association with overall satisfaction for GPs was the opportunity to use their abilities with more than 40% of explained variance. While the performance of GPs depends on their own skills and capabilities, it also requires sufficient time and support from non-physician staff [24]. German GPs have the highest rate of patient contacts per week compared to other countries [11]. Enhancing the role of non-physician staff might improve the quality of patient care [25]. However, it has been observed that a higher percentage of staff members per practice could also increase the workload of physicians [26].

### Strengths and limitations

A main strength of our study is that there has been little research on the job satisfaction of both GPs and non-physician staff in the same primary care practices until now [15]. However, our sample may not be representative for all primary care practices in Germany because we only involved practices which were willing to participate in a quality management system and we had less solo practices in our sample than the national average suggests [12]. In 2005, the German government stipulated that health care providers should implement a system of annual assessment of quality management [18]. However, no essential health policy system changes could affect the results during the period of data collection. Furthermore, a systematic bias could be suspected because there are large differences between GPs and non-physician staff regarding the socio-demographic characteristics. However, it is not possible to adjust for these differences in our analysis. Additionally, it was not feasible to pair the data of GPs and non-physician staff on practice level since the number of GPs and non-physician staff varied among the practices. A strong aspect was the availability of large numbers of data on German primary care practices including data from staff. We used internationally validated measures for the evaluation of job satisfaction by physicians and non-physicians. In addition, this was an exploratory study; p values should be interpreted carefully. Significant results might be due to chance and will need to be confirmed in further targeted studies.

## Conclusions

Non-physician staff members rated their job satisfaction higher than GPs. For GPs and non-physician staff different elements were relevant for the evaluation of their overall job satisfaction. On the one hand, for GPs the opportunity to use their abilities had the strongest association with job satisfaction, whereas for non-physician staff, recognition of their role and performance was most important. However, in both cases, this was offset by the perception of poor income. The findings of this study will be helpful for further activities to improve the working conditions of GPs and non-physician staff from different perspectives. Non-physician staff should be supported in their role implicating recognition and appreciation for performance in practice. Moreover, GPs need support through *Continuing Professional Development *and a team to enable them to use their abilities. In these premises, further research might focus on whether measures to improve the factors we found, really contribute positively to job satisfaction prospectively.

## Competing interests

BB and SW are employed by the AQUA-Institute which disseminates EPA in Germany. JSz is its director and stockholder.

Other authors: No conflict of interest declared.

## Authors' contributions

KG, SC and JSz initiated and designed the study. SW and BB coordinated the study. KG carried out data analysis and wrote the manuscript. All authors read earlier versions of the manuscript, provided critical comments and approved the final manuscript.

## Pre-publication history

The pre-publication history for this paper can be accessed here:

http://www.biomedcentral.com/1471-2296/12/137/prepub
